# The Fast Track for Intestinal Tumor Cell Differentiation and In Vitro Intestinal Models by Inorganic Topographic Surfaces

**DOI:** 10.3390/pharmaceutics14010218

**Published:** 2022-01-17

**Authors:** Matteo Centonze, Erwin J. W. Berenschot, Simona Serrati, Arturo Susarrey-Arce, Silke Krol

**Affiliations:** 1 Laboratory for Personalized Medicine, National Institute of Gastroenterology, “S. de Bellis” Research Hospital, Castellana Grotte Via Turi 27, 70013 Bari, BA, Italy; matteo.centonze@irccsdebellis.it; 2 Mesoscale Chemical Systems, MESA+ Institute, University of Twente, P.O. Box 217, 7500 AE Enschede, The Netherlands; j.w.berenschot@utwente.nl; 3 Nanotechnology Laboratory, IRCCS Istituto Tumori “Giovanni Paolo II”, Viale Orazio Flacco 65, 70124 Bari, BA, Italy; simonaserrati@hotmail.com

**Keywords:** HT29 differentiation, lectins, topographic surfaces, Paneth cells, in vitro intestinal model

## Abstract

Three-dimensional (3D) complex in vitro cell systems are well suited to providing meaningful and translatable results in drug screening, toxicity measurements, and biological studies. Reliable complex gastrointestinal in vitro models as a testbed for oral drug administration and toxicity are very valuable in achieving predictive results for clinical trials and reducing animal testing. However, producing these models is time-consuming due to the lengthy differentiation of HT29 or other cells into mucus-producing goblet cells or other intestinal cell lineages. In the present work, HT29 cells were grown on an inorganic topographic surface decorated with a periodic pattern of micrometre-sized amorphous SiO_2_ structures for up to 35 days. HT29 cells on topographic surfaces were compared to undifferentiated HT29 in glucose-containing medium on glass or culture dish and with HT29 cells differentiated for 30 days in the presence of methotrexate (HT29-MTX). The cells were stained with Alcian blue for mucus, antibodies for mucus 2 (goblet cells), villin (enterocytes), lysozyme (Paneth cells), and FITC-labeled lectins to identify different cells, glycomic profiles, and cell features. We observed that HT29 cells on topographic surfaces showed more similarities with the differentiated HT29-MTX than with undifferentiated HT29. They formed islands of cell clusters, as observed for HT29-MTX. Already after 2 days, the first mucus secretion was shown by Alcian blue stain and FITC-wheat germ agglutinin. After 4–6 days, mucus was observed on the cell surface and in the intercellular space. The cell layer was undulated, and in 3D reconstruction, the cells showed a clear polarisation with a strong actin signal to one membrane. The lectins and the antibody-staining confirmed the heterogeneous composition of differentiated HT29 cells on topographic surfaces after 6–8 days, or after 6–8 days following MTX differentiation (30 days).

## 1. Introduction

Three-dimensional (3D) cell culture allows for a clearer understanding of disease mechanisms, e.g., for inflammatory bowel disease or colorectal cancer. It provides more reliable results in drug screening, especially for orally administered drugs and their uptake by the gut [1,2,3,4,5,6,7] due to the presence of mucus and different cells and cell layers. However, it is still not yet frequently used for routine lab work, as producing realistic complex intestinal in vitro models is time-consuming. The differentiation of, e.g., multipotent stem cells or poorly differentiated multipotent (stem cell-like) cells, such as HT29, from human colonic mucosa adenocarcinoma is a lengthy and costly process [6,8].

The standard procedure is the maintenance of the cells for several weeks under a constant regime of different chemical agents (sodium butyrate, suramin, trehalose, 12-0-tetradecanoylphorbol13-acetate, galactose) or in glucose-free medium to induce the systematic differentiation of HT29 [9]. In the presence of glucose, the HT29 cells remain undifferentiated [10]. Differentiation requires between 21 days and 1 month. Other researchers have induced HT29 differentiation using forskolin or colchicine [10] or by long-term exposure to low doses of methotrexate (MTX) [11]. Depending on the conditions, the resulting differentiated HT29 cells present a mixture of mucus-producing goblet cells and an absorptive microvilli-presenting enterocyte-like phenotype. In co-culture with Caco2 cells, a cell line similar to enterocytes, HT29-MTX cells produce a complex intestinal in vitro model, with enterocytes, goblet cells, tight junctions, and a mucus layer in 2D [12,13].

Embedded in a gel matrix, either Matrigel or other hydrogels, and induced by differentiation factors, intestinal stem cells form 3D organoids or hollow enteroids, a valuable tool for studying viral and bacterial intestinal infections [2,14]. One major limitation of gastrointestinal organoids is that the mucus is located in the interior, requiring microinjection to study transport processes from the lumen to the enterocytes [15] or an additional step to open up the enteroid into a 2D cell layer.

This work explores a new cell culture platform with periodically organised micrometre-sized amorphous SiO_2_ structures as a topographic surface that differentiates HT29 cells without additional agents and in the presence of glucose. The resulting cell layers were analysed by lectin assays and immunohistochemistry for the presence of mucus and different intestinal cell lineages such as goblet cells, enterocytes, and Paneth cells. The resulting cells were compared to undifferentiated HT29 cells and HT29 differentiated for 30 days in the presence of MTX (HT29-MTX).

## 2. Materials and Methods

### 2.1. Topographic Surfaces

**Figure 1:**
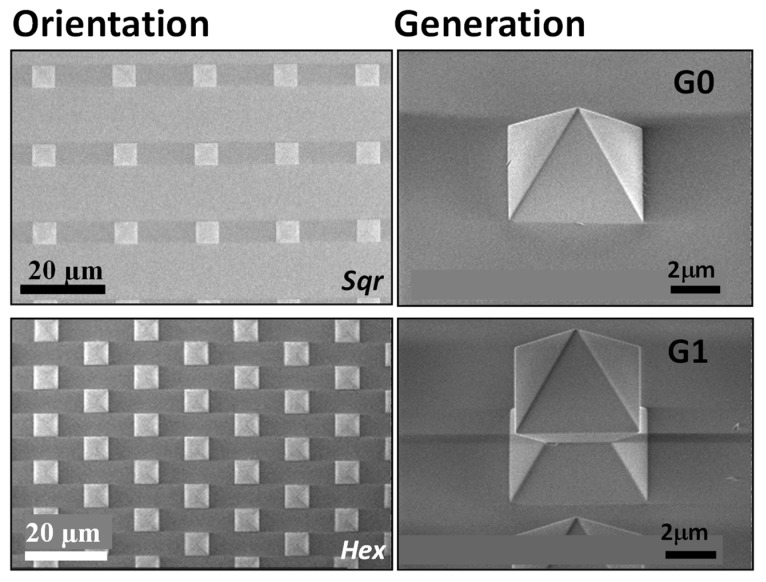
SEM images of the topographic structures increasing in self-similarity and complexity between generations.

The topographical surfaces were a gift from Encytos B.V. (Enschede, The Netherlands). The structure geometries (G) are shaped in a pyramid (G0) or octahedron (G1), as shown by SEM images in Figure 1, and consist of amorphous SiO_2_.

The periodic pattern follows either a hexagonal (*Hex*) or square (*Sqr*) orientation. Each structure has specific dimensions (structure-to-structure interspace and height), presented in Figure S1.

### 2.2. Cell Culture

Human colon adenocarcinoma HT29 cells (ATCC^®^ HTB38™; LGC Standards S.r.l., Sesto San Giovanni, Italy) were cultured in Dulbecco’s Modified Eagle Medium (DMEM) supplemented with 10% foetal bovine serum (FBS), sodium pyruvate, antibiotic–antimycotic and Hepes (all: Thermo Fisher Scientific, Waltham, MA, USA). Cells were cultured at 37 °C in humidified atmosphere containing 5% CO_2_. The medium was changed every three days.

To differentiate HT29 cells in traditional 2D cell culture, the cells were exposed to methotrexate (MTX). Depending on the MTX concentration, the resulting cells change from enterocytes to mainly mucus-secreting cells [11]. According to the protocol of Lesuffleur et al. [11], in this study, the HT29 cells were incubated for 30 days in the presence of 10^−5^ M MTX to induce mainly goblet cells.

#### Culture on Topographic Surfaces

Topographic surface substrates (1 cm × 1 cm) were placed in 6-well plates and sterilised by irradiation with UV-light in the laminar flow hood for 1 h. HT29 cells were trypsinised and resuspended in complete DMEM medium at the concentration of 4 × 10^5^ cells/mL. Then, 50 μL of cell suspension (containing 2 × 10^4^ cells) were seeded on the sterilised substrates. First, the cells were incubated for at least 4 h at 37 °C and 5% CO_2_ without additional medium allowing them to attach exclusively onto the topographically structured surfaces.

Then, the substrates were covered with complete medium and placed in the incubator, changing the medium every 3 days. At day 8 and day 11, one substrate for each HT29 sample was fixed for 10 min with 4% paraformaldehyde in phosphate-buffered saline (PBS) at pH = 7.4, respectively. Each experiment was performed in duplicate or triplicate.

Respective control cells were grown on treated 24-well plates (Corning Cellbind Surface; Corning Inc., New York, NY, USA) or glass surfaces. These cells will be refered to as traditional 2D culture.

### 2.3. Imaging

#### 2.3.1. Trypan Blue Exclusion Assay for Dead Cells

Cells were washed with PBS to eliminate residual serum proteins, which could be stained by trypan blue. A solution of 0.2% trypan blue was added to the cells. After ~3 min of incubation at room temperature, the cells were washed with PBS and fixed with 4% para-formaldehyde (PFA) for 10 min.

#### 2.3.2. Alcian Blue Staining for Mucus

For the visualisation of mucus, the cells were fixed with Methacarn according to the protocol of Xavier et al. [16]. The cells were washed with PBS and fixed in cold Methacarn (60% methanol, 30% chloroform, 10% acetic acid) for 15 min at 4 °C [17,18]. Cells were then washed in a 3% acetic acid solution in H_2_O, stained with Alcian blue 8XG for 15 min at RT, and washed with water.

While the chloroform partially dissolved the surface of the culture dish for the control cells (Figure S1), the silica surface of the templates was inert. Therefore, the control experiments with HT29-MTX and HT29 were performed on glass surfaces using microscopic cover slips.

#### 2.3.3. Fluorescence Microscopy

The epifluorescence images were acquired with a basic Nikon eclipse Ti2 microscope (Nikon, Tokyo, Japan). For confocal microscopy, the system was additionally equipped with a Nikon C2 laser unit with 4 laser lines (405, 488, 561 and 640 nm) and was able to record images with a resolution of up to 2048 × 2048 μm pixels. The microscope was controlled by the NIS-Elements software.

#### 2.3.4. Staining Protocols

The visualisation of HT29 cells with antibodies (MUC2-Mucin 2, Oligomeric Mucus/Gel-Forming; villin; lysozyme) and lectins (PNA-peanut agglutinin; UEA-*Ulex Europaeus* agglutinin; WGA-wheat germ agglutinin) labeled with different fluorophore required a serial incubation. The incubation conditions are summarised in Table 1.

Briefly, fixed HT29 cells were permeabilised with 0.1% Triton X-100 in PBS for 10 min and washed with PBS. Cells were then incubated with 1% BSA in PBS to block unspecific binding of the antibodies for 30 min and subsequently for 2 h at room temperature with the specific primary antibody or FITC-labeled agglutinin diluted in 1% BSA in PBS. All agglutinins (PNA: FL-1071; UEA: FL-1061; WGA: FL-1021) were purchased from Vector Laboratories (DBA ITALIA S.R.L., Segrate, Italy) and used at a concentration of 2 μg/mL. All primary antibodies (MUC2: ab11197; Villin: ab130751, Lysozyme: ab108508) were purchased from Abcam (Cambridge, MA, USA) and diluted 1:200. For visualisation of the actin filaments of the cytoskeleton, the cells were incubated with 5 μg/mL Phalloidin-Tetramethyl-rhodamine B isothiocyanate (TRIC; P2141, Sigma-Aldrich, Milan, Italy). After incubation with primary antibodies, the cells were rinsed with PBS and incubated for 1 h at room temperature with an appropriate fluorescent dye-labeled secondary antibody (goat anti-rabbit and goat anti-mouse IgG, Alexa fluor plus 594, (both: Thermo Fisher Scientific, Waltham, MA, USA), diluted 1:500. After three washes with PBS, the adherent cells were covered with 4′,6-diamidino-2-phenylindole (DAPI)-supplemented antifade mounting medium (VECTASHIELD, (DBA ITALIA S.R.L., Segrate, Italy) for visualisation of nuclei, followed by visualisation in fluorescence microscopy (NIKON Eclipse Ti2; Nikon, Tokyo, Japan).

#### 2.3.5. Image Analysis

The ratio of the antibody-positive cells to the total number of cells was calculated by means of the plugin colour pixel counter of FIJI [19], and the percentage of green- or red-coloured pixels was determined. First, the images were corrected until the background was 0 using the NIS software from Nikon (Nikon, Tokyo, Japan). The total number of coloured pixels was counted by setting the threshold to 10, while for the antibody-stained cells, the threshold was set to 100–150. For each data point, 3–7 images were analysed.

## 3. Results

HT29 cells were seeded in glucose-containing medium on topographic structures of G0 (pyramids), and G1 (octahedrons) after UV sterilisation but without any pre-treatment. As no difference in the cellular behaviour was found on hexagonal- or square-oriented surface patterns (Figure S1 in the Supplementary Material), in the following, the data are presented for cells on either Gx*Hex* or Gx*Sqr*. The results on the topographic surfaces were compared with HT29 cells either grown in the 2D cell culture on glass or pre-treated culture dishes. Moreover, the cell morphology and biomarker expression of HT29 cells on topographic surfaces were compared to HT29 cells differentiated for 30 days in the presence of MTX (10^–5^ M) (HT29-MTX) and then grown on glass or in culture dishes. For some experiments, the experiments on pre-treated 24-well plates had to be excluded, as the chloroform-containing Methacarn fixative used to preserve the mucus damaged the culture dish surface (Figure S2).

### Mucus Production

**Figure 2:**
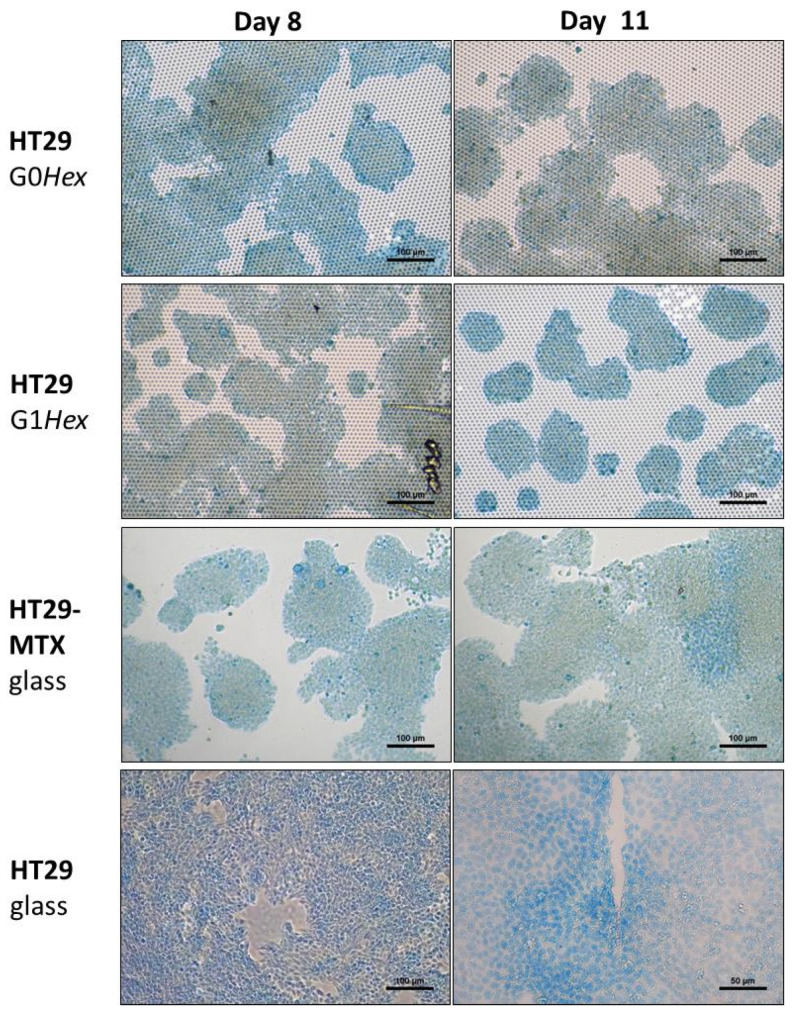
Alcian blue staining of mucus released by HT29 cells, as well as MTX-differentiated HT29 cells on glass and topographic surfaces (G0*Hex*, G1*Hex*). Scale bars: 100 μm, except for HT29 (11 days, glass), where scale bar: 50 μm. Number of independent experiments: *n* = 4.

Methacarn-fixed HT29/-MTX cells on glass and on the topographic surfaces were stained for mucus with Alcian blue on day 8 and on day 11 (Figure 2).

On day 8, the HT29 cells on the topographic surfaces were strongly positive by Alcian blue staining, comparable to the MTX-differentiated cells (Figure 2). Additionally, the morphology of the cell clusters in islands was similar in MTX-differentiated cells and on the topographic surfaces. Conversely, the HT29 cells grew in a continuous layer in the presence of glucose-containing medium.

To understand on which day the mucus release started, the cells were followed on day 1, 2, 4, 8, and 11 (Figure 2, Figures S3 and S4). On day 1, only single cells were observed (Figure S3); therefore, no mucus test was performed. On days 2 and 4, HT29 on topographic surfaces showed a strong Alcian blue signal (Figure S4). The HT29 cells in glucose-containing medium on culture-treated 24-well plates showed a low bluish staining, but this seems to be more localised in the nucleus. In fact, Alcian blue was also used as nuclear staining [20]. On days 8 and 11, the Alcian blue signal was stronger, and the difference in differentiated HT29-MTX cells as well as HT29 cells on the topographic surfaces (islands) and the undifferentiated HT29 cells on glass (uniform cell layer) was more obvious (Figure 2).

The growth behaviour of HT29 on topographic surfaces or in traditional 2D culture differed already on day 1 (Figure S3). The HT29 cells in traditional 2D culture (24-well plate) in the presence of glucose were homogenously distributed, while HT29 cells on topographic surfaces started forming loosely attached clusters. On day 4, on topographic surfaces, the patches had grown to large islands of densely packed cells, while in traditional culture, they formed a uniform layer. On day 8, cells with a heterogeneous morphology were observable on the topographic surfaces, while on the culture dish, the cell morphology was more homogenous.

**Figure 3:**
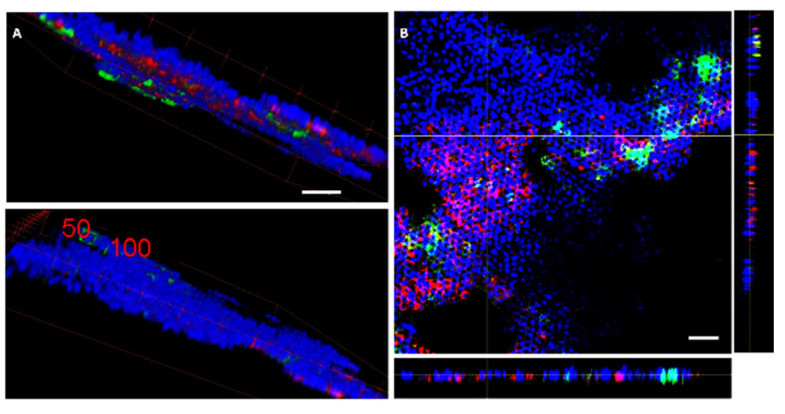
HT29 cells were seeded for 15 days on G0*Hex* surfaces in glucose-containing medium without MTX. The cells were fixed with PFA and stained with TRITC-phalloidin for actin filaments (red), FITC-UEA for MUC2 on goblet cells (green), and DAPI for the nuclei (blue). By confocal microscopy, a z-stack was recorded and visualised in 3D volume view. (**A**) Front and back of the cell layer (see also supporting video 1). (**B**) Image on the focal plane of the cell layer in x-y, x-z, and y-z direction. Number of independent experiments: *n* = 2. Scale bars: 50 μm.

Phalloidin-staining was used to visualise the actin filaments. In Figure 3A, in the front view (top lane), it is obvious that the red signal is mainly localised there, while in the back view of the same cells (bottom view in Figure 3A), almost no red signal is visible.

This inhomogeneous distribution of actin in cells indicates cell polarisation, and hence HT29 differentiation. Undifferentiated cells are not polarised, and have a homogenous distribution of the actin or even bundles of stress fibers. Differentiated cells such as goblet cells and enterocytes are polarised [14,21,22] and have an accumulation of actin of the luminal side. In the 3D reconstruction of a z-stack recorded by confocal microscopy with a phalloidin staining for actin, we observed a clear localisation of the actin signal (Figure 3A). The cells were co-stained with DAPI for the nucleus and FITC—UEA. UEA binds to MUC2-positive mucus released by goblet cells [23] and to fucose, found on M cells in Peyer’s patches in mouse intestine [24,25,26] and on intestinal mucus [27]. Fucose plays an important role in intestinal health and cross-talk with the microbiota [28]. Moreover, it has been shown that fucose is localised in brush cells, which are positive for UEA, in contrast to enterocytes [29].

In the x-z and y-z view in Figure 3B, the cell layer appears not entirely flat, but undulated (see y-z image in Figure 3B). This is in agreement with observations for 2D cell models with organoids on filters [14].

**Figure 4:**
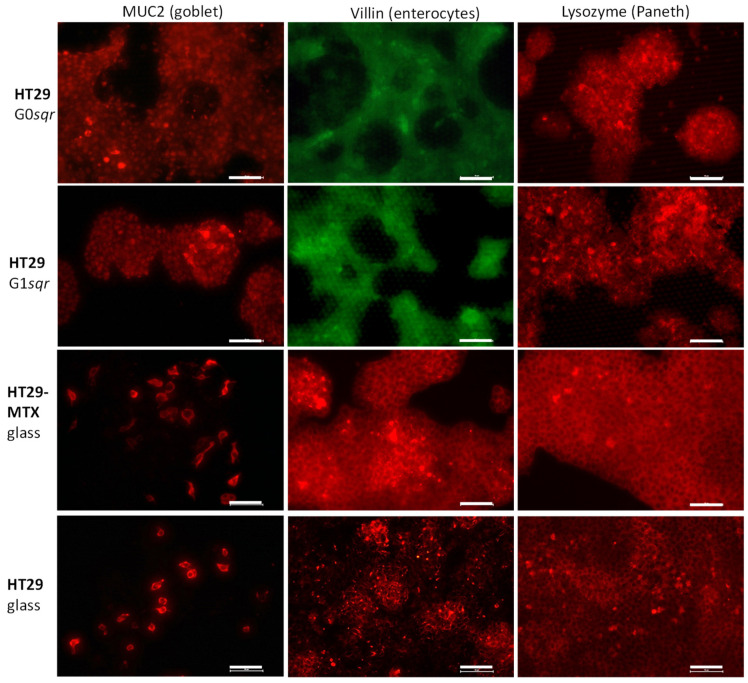
Epifluorescence of HT29, as well as HT29-MTX cells grown on different substrates for 8 days, immunolabelled by antibodies for MUC2, villin, lysozyme and then stained TRITC-(red) or FITC-(green) labeled secondary antibodies. Number of independent experiments: *n* = 2. Scale bar: 50 μm.

The HT29 cells on topographic surfaces, glass, and in culture dishes, as well as the MTX-differentiated HT29, were stained with antibodies (Figure 4) indicative for cell biomarkers such as MUC2 (intestinal goblet cells [30]), villin (enterocytes, brush border cells), and lysozyme (Paneth cells).

MUC2 is frequently used to confirm the successful differentiation of HT29 to goblet cells [12,31]. Villin is a biomarker for enterocytes, involved in the assembly and maintenance of microvilli in polarised epithelial cells [14,21,22]. Lysozyme was previously used to identify Paneth cells [5], terminally differentiated crypt cells which are involved in immune defense by producing bactericidal molecules, i.e., lysozyme [32]. These studies are supported by staining with different FITC-labeled lectins (UEA, WGA, PNA; Figure S5).

The immunofluorescence images were analysed for the percentage of intensely coloured pixels considered antibody (Ab)-positive, while the cells were identified by a lower signal against a black background. The images were analysed by the FIJI (colour pixel counter). The intensity threshold for Ab-positivity was 100, the background was corrected to be 0, while an intensity for >10 was considered as a cell. The calculated data are summarised in Table 2.

In the immunofluorescence images in Figure 4, for all cell systems, few cells show a strong MUC2 signal (first row). This is in agreement with the signal found for UEA, which can also bind to MUC2, and showed only small areas with elevated fluorescence intensity (second row in Figure S5). As both molecules bind to MUC2, we performed co-staining with UEA and MUC2 on HT29 after 8 days on the topographic surfaces (Figure S6A). Our experiments showed no co-localisation of the red signal for MUC2 antibodies staining strongly single cells and the more diffuse green signal for FITC-UEA. This finding is in contrast to Gouyer et al. [23], who found a co-localisation of UEA and MUC2 antibodies. The reason for this is not completely clear, but the UEA signal seems to be located on released mucus, while the intercellular localisation of the MUC2 antibody seems to indicate that it stains the goblet cells themselves.

Interestingly, image analysis indicates that in cells grown in traditional 2D culture, there are more cells with MUC2 expression than for cells grown on topographic surfaces (Table 2).

The villin signal is present in most cells, with some cells in clusters that show a higher fluorescence intensity (Figure 4, middle row). The image analysis showed that the villin content was higher in 2D cell culture than on the topographic surfaces on day 4 and 8. Only on day 11 on the topographic surfaces did the villin content increase, especially for the HT29 cells grown on G0 pyramidal structures (Table 2).

WGA binds to collagen type 1 of the extracellular matrix and in fibrotic tissue [33] or to glycans on the mucus layer in the intestine [27]. It was also used for the detection of sialic acid and acetylglucosamine present on M cells in a Caco2/Raji B cells [34,35]. The signal for WGA (Figure S5; first row) mainly confirms the observations of the mucus staining with Alcian blue (Figure 2). There is a background of mucus and extracellular matrix with few cells, which showed high intensity both for Alcian blue or WGA.

The most interesting observation was made for the lysozyme staining. It was observed that the cells differentiated on the topographic surfaces have a higher content of lysozyme-containing cells than the 2D cultured cells (Figure 4, last row), which is also confirmed by the image analysis (Table 2). The lysozyme is localised in vesicular structures in the cells (Figure S7), which is typical for Paneth cells. It was found that lysozyme is stored in secretory antibacterial granules in Paneth cells in the Lieberkuehn crypt in the small intestine [36].

PNA binds with good specificity in patients with inflammatory bowel disease and colon cancers to the exposed Thomsen–Friedenreich (TF) oncofetal carbohydrate antigen (galactoseβ1-3N-acetylgalactosamineα) [37,38,39,40] in the diseased areas. In our study, the signal for PNA showed multiple vesicular-like structures in sparsely distributed cells in cell clusters on all tested surfaces (Figure S5, last row). We also stained for co-localisation of PNA and lysozyme (Figure S6B), but as expected, there is no co-localisation of the green PNA signal and the red fluorescence signal of lysozyme.

## 4. Discussion

In summary, the topographic surfaces with amorphous SiO_2_ pyramids or octahedrons differentiate HT29 [41], an undifferentiated, multipotent colon carcinoma cell line in a shorter time and more cell lineages than with traditional inducer techniques. While chemical inducers or glucose-deprivation usually foster differentiation into one intestinal cell lineage after a massive cell death of all HT29 cells not adapted to the experimental conditions, the topographic surfaces allow the differentiation of the cells without a medium change or any additives from 2D pre-culture to differentiation.

In comparison with HT29-MTX cells and HT29 cells on glass in traditional 2D culture, the 2D cells showed more MUC2-positive cells than cells grown on topographic surfaces. However, the appearance of a continuous mucus layer on day 2 on Ht29 cell on topographic surfaces (Figure S4) confirmed by Alcian blue staining supports the presence of pre-existing goblet cells.

The temporal development for the other cell markers such as lysozyme or villin on the topographic surfaces indicates that the cell differentiation initiates between 4 and 8 days. While usually the differentiation is induced by glucose deprivation or chemical inducers tailoring the differentiation into distinctive lineages, in our case, the topographic structures and their distance might play a role. That topography and the material influence the response of cells not only in terms of their mechanical properties but also with respect to their genetic expression profile, as shown previously by Jang et al. [42]. Mydin et al. [43] showed that HT29 cells on upright titanium dioxide nanotubes were highly positive for CK8. CK8-keratin, type II cytoskeletal 8 also plays a role in mechanosensitive cell differentiation. However, no further biomarkers were studied to confirm the differentiation.

HT29 cells in traditional 2D cell culture and in the presence of glucose on pre-treated plastic surfaces usually remain undifferentiated [10,41]. They grow in a continuous cell layer (Figure 3 and Figure S3) and might partially differentiate upon reaching post-confluence [10,12]. In contrast, HT29 differentiated for 30 days in the presence of methotrexate followed by 2D cell culture standard medium as well as HT29 on the topographic surfaces cultured for 6–8 days show many similarities in morphology and growth behaviour. They grew in extended islands as shown by us (Figure 3, Figures S3 and S4) and others [44]. The differentiated HT29 cells on the topographic surfaces formed slightly undulated monolayers, as in the intestine. The heterogeneous distribution of the actin (Figure 4) indicates a polarisation of HT29 cells on the templates and hence further confirms the differentiation.

It is interesting that the differentiating HT29 colon cancer line forms a 2D cell layer. It has previously been observed that primary tumour cells with cancer-associated fibroblasts and hepatocellular carcinoma cell lines such as HLF form on the same G1 spheroids [45]. It seems that on the topographic substrates, the cell growth is supported in its natural form. The possibility of forming cell sheets and support seems to be crucial, as Goodwin et al. [46] observed cell growth and spheroid formation for HT29 cells in the rotating wall vessel when they added cyclodex-3 type 1, collagen-coated dextran 175-μm microparticles.

The lysozyme immunofluorescence indicates that the topographic surfaces differentiate HT29 cells into Paneth cells, located in the proliferative crypt region of the intestine in the vicinity of the Lgr5 stem cells and play an important protective role in the immature small intestine [32,36]. This region presents a less mature region of the intestine, which might also explain the presence of PNA positivity. This could also explain the relatively lower presence of MUC2 as compared to the MTX-differentiated HT29 cells. This profile of differentiated cells on topographic surfaces presents an interesting in vitro model for inflammatory bowel disease (IBD), which is characterised by PNA binding Thomsen–Friedenreich antigen-exposed cells [47] and Paneth cells, which play a key role in IBD [48,49].

## 5. Conclusions

Periodically, patterns of inorganic amorphous SiO_2_ pyramidal and octahedral structures induce a rapid differentiation in HT29 cells. This novel growth platform reduced the time from 30–40 days for differentiation with MTX to just 6 days. The differentiated HT29 cells develop into a complex in vitro intestinal model, as they start to produce mucus at day 2 and are strongly positive for lysozyme indicator for Paneth cells. To our knowledge, this is the first time that inorganic surfaces with a periodic microstructure patterning have been used to induce differentiation of HT29 to Paneth cells in only 6 days without the addition of any other factors or pre-treatment.

## 6. Patents

The following patent was filed resulting from the work reported in this manuscript: PCT/NL2021/050409.

## Figures and Tables

**Table 1:** Serial staining protocol.

Fixative	1st Step	2nd Step	3rd Step	4th Step
PFA	Fixation	TRITC-Phalloidin	FITC-UEA	DAPI
	Propidium iodide	Fixation	FITC-UEA	DAPI
	Fixation	Alcian blue		
	Fixation	Antibody (MUC2; lysozyme; villin)		
Methacarn	Trypan blue	Imaging	Fixation	Alcian blue
	Fixation	FITC-WGA/-PNA/-UEA	Imaging	Alcian blue
	Fixation	FITC-PNA	DAPI	
	Fixation	FITC-lectin	DAPI	Antibody (MUC2/lysozyme/villin)

PFA: para-formaldehyde.

**Table 2:** Image analysis of immunofluorescence on HT29 cells as well as HT29 differentiated with MTX on different surfaces.

Cell Culture (Days)	MUC2 %	Villin %	Lysozyme %
HT29 on 24-well plate *	5.84 ± 3.50	4.67 ± 0.21	1.12 ± 0.69
HT29-MTX on 24-well plate *	8.67 ± 4.76	4.04 ± 2.78	0.18 ± 0.13
HT29 on G0 (4 days)	0	0.35 ± 0.47	0.63 ± 1.10
HT29 on G0 (8 days)	1.19 ± 1.45	0.38 ± 0.34	11.17 ± 7.65
HT29 on G0 (11 days)	2.14 ± 1.40	6.26 ± 2.00	12.34 ± 9.25
HT29 on G1 (4 days)	0.01 ± 0.02	0.14 ± 0.09	0
HT29 on G1 (8 days)	2.42 ± 1.43	0.27 ± 0.32	6.99 ± 4.60
HT29 on G1 (11 days)	0.57 ± 0.67	1.92 ± 1.58	10.97 ± 2.12

%: pixels (Ab) considered positive > 100/pixels (cells) considered positive > 10. Images (*n* = 5–7) were analysed with FIJI (colour pixel counter). * The image analysis was performed either on day 8 or on day 11 or on both days. No significant difference in expression was found between the two days.

## Data Availability

All data relevant to the publication are included.

## Supplementary Materials

The following are available online at https://www.mdpi.com/article/10.3390/pharmaceutics14010218/s1, Figure S1: Light microscopic images comparing the cell growth of HT29 cells after 4 days on G0 and G1 with square (Sqr) and hexagonal (Hex) orientation. The corresponding pit width and the height of the structures can be seen in the table. Figure S2: Damage on culture dish due to Methacarn fixation; Figure S3: Time lapse experiment on topographic surfaces versus traditional 2D culture (24-well plate). Figure S4: Light microscopic images of Methacarn-fixed and Alcian blue-stained HT29 cells on day 2 and 4. Figure S5: Epifluorescence of HT29 as well as HT29-MTX cells on different substrates stained with FITC-lectins (UEA—Ulex Europaeus agglutinin; WGA-Wheat Germ agglutinin, PNA-Peanut agglutinin). Figure S6: Immunofluorescence images of co-localisation experiment of glycans with goblet or Paneth cells. Figure S7: Magnified images of cell clusters on topographic surfaces stained for lysozyme.
