# Supplementary material for: The Fast Track for Intestinal Tumor Cell Differentiation and In Vitro Intestinal Models by Inorganic Topographic Surfaces
Source: Pharmaceutics. 2022 Jan 17;14(1):218. doi: 10.3390/pharmaceutics14010218 (PMC8781367; doi:10.3390/pharmaceutics14010218)
Supplement: Supplementary file 1 [file pharmaceutics-14-00218-s001.zip › pharmaceutics-1500077-supplementary.pdf]

pharmaceutics

MDPI

Supplementary Materials

The Fast Track for Intestinal Tumor Cell Differentiation and In Vitro Intestinal Models by Inorganic Topographic Surfaces

Matteo Centonze 1, Erwin J. W. Berenschot 2, Simona Serrati 3, Arturo Susarrey-Arce [2],* and Silke Krol [1],*

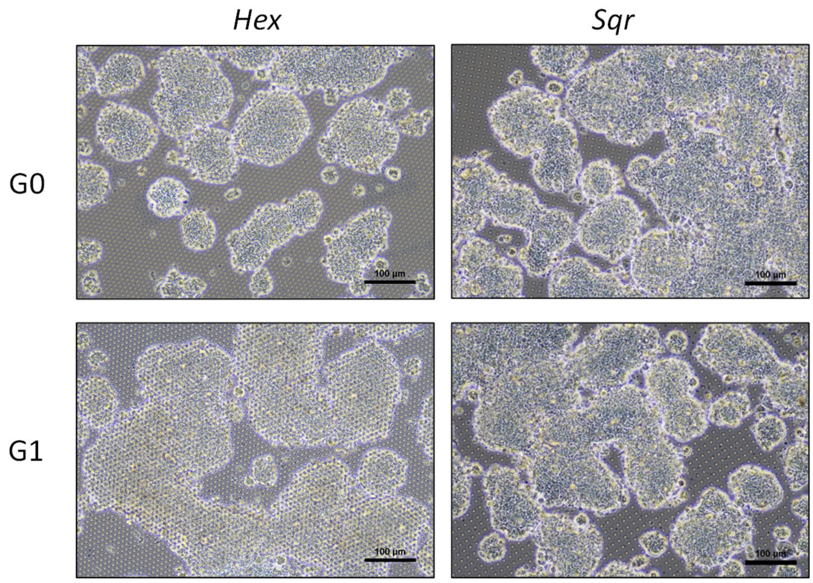

Geometrical Parameters		
	**Height**	**Interspace**
**G0*Sqr***	4.03	20.0
**G1*Sqr***	8.10	19.9
**G0*Hex***	4.2	12
**G1*Hex***	8.0	11.9

Figure S1. Light microscopic images comparing the cell growth of HT29 cells after 4 days on G0 and G1 with square (*Sqr*) and hexagonal (*Hex*) orientation. Scale bars are 100 µm. The corresponding pit width and the height of the structures can be seen in the table.

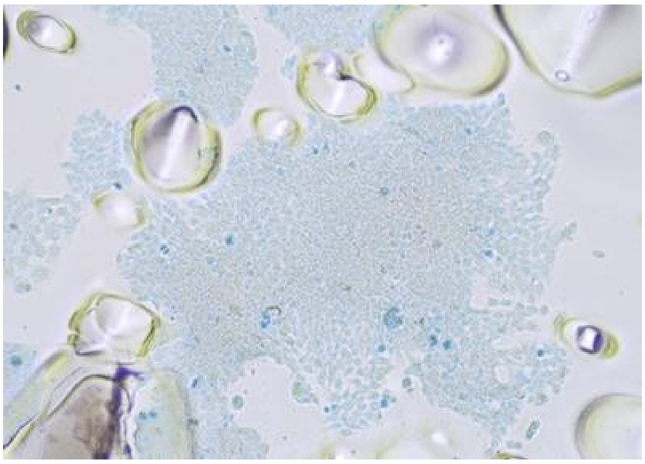

Figure S2. Damage on culture dish due to Methacarn fixation. Evident is the damage on the plastic surface of the 24-well plate due to chloroform interrupting the previously homogenous cell layer. Magnification: 4×.

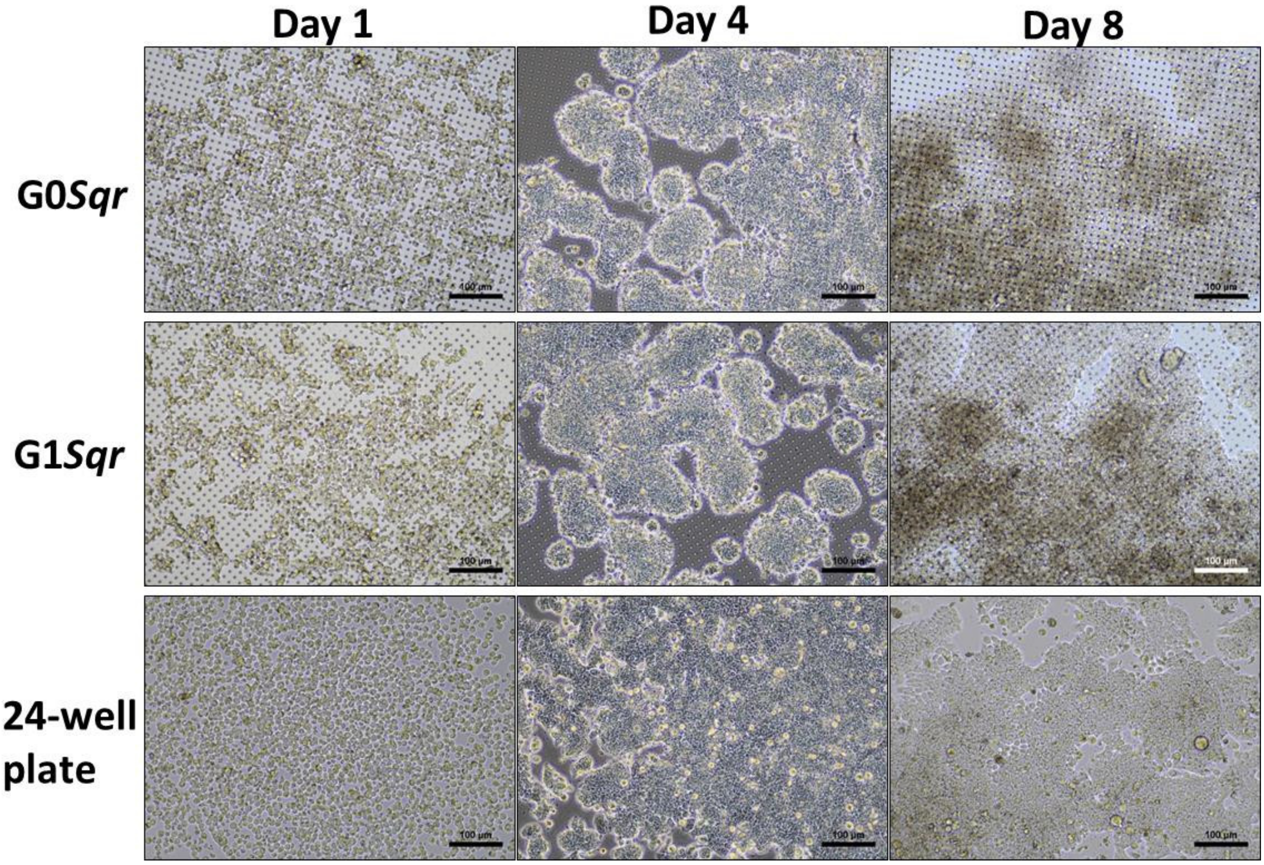

Figure S3. Time lapse experiment on topographic surfaces versus traditional 2D culture (24-well plate). Light microscopic images of HT29 cells seeded on G0 and G1 topographic substrates and on pre-treated 24-well plates at day 1, 4 and 8. Scale bars: 100 µm.

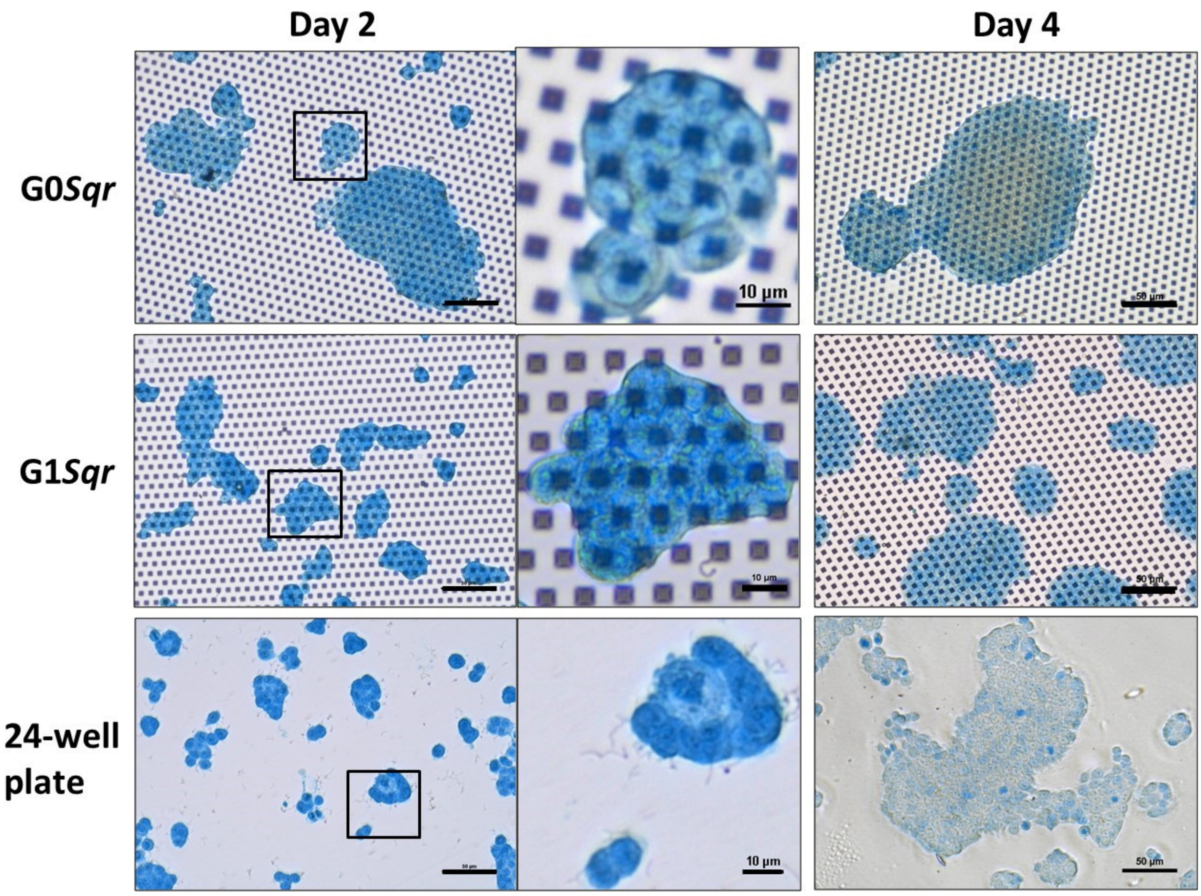

Figure S4. Light microscopic images of Methacarn-fixed and Alcian blue-stained HT29 cells on day 2 and 4. The images in the middle row are the magnification of the islands in the frames in the images on day 2. Scale bars (day 2, 4): 50 µm. Scale bar (magnification): 10 µm.

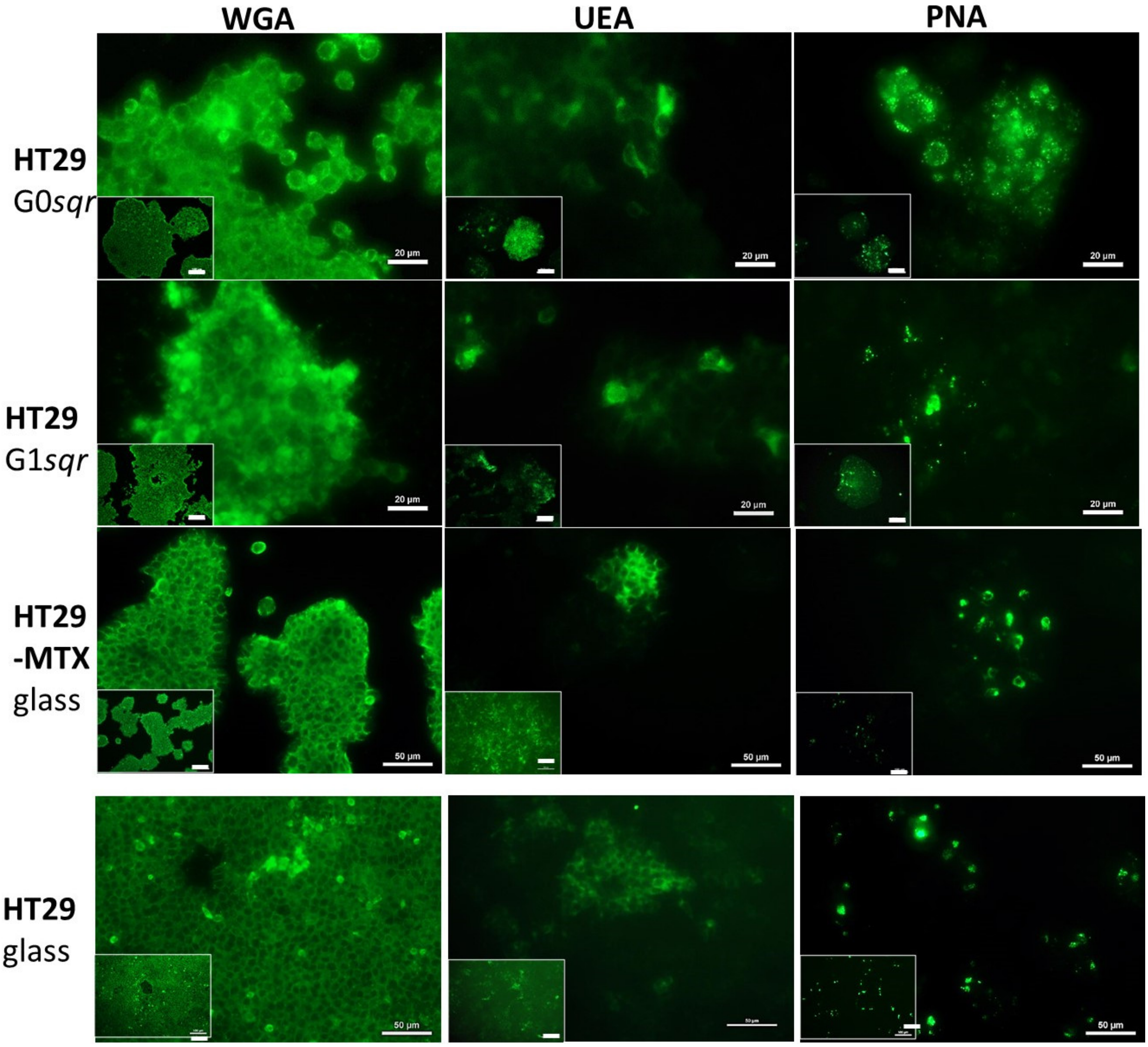

Figure S5. Epifluorescence of HT29 as well as HT29-MTX cells on different substrates stained with FITC-lectins (UEA- *Ulex Europaeus* agglutinin; WGA-Wheat Germ agglutinin, PNA-Peanut agglutinin). Scale bar (inset): 100μm. Scale bar (upper 2 panels): 20 μm; (lower 2 panels): 50 μm.

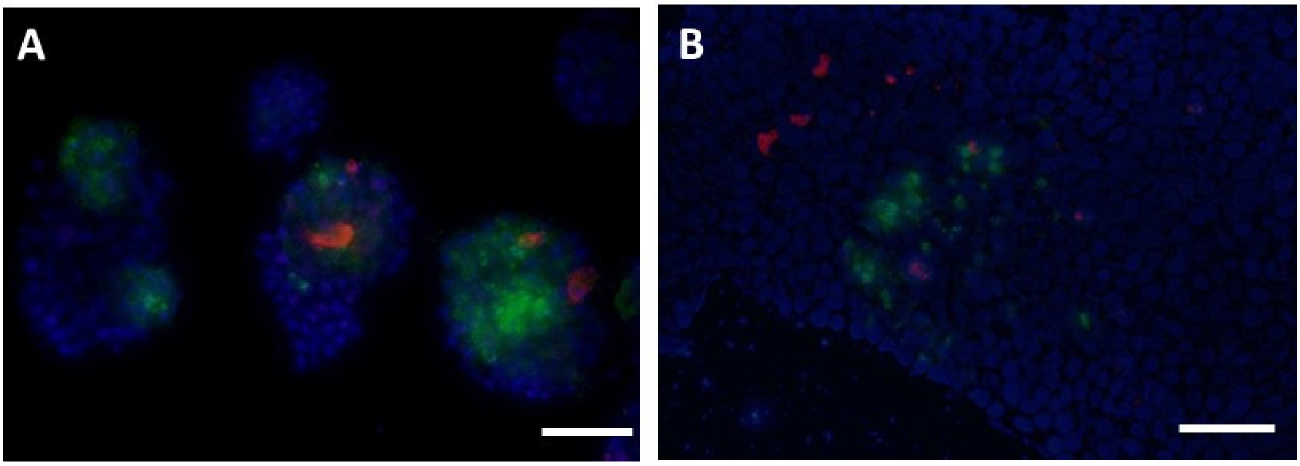

Figure S6. Immunofluorescence images of co-localization experiment of glycans with goblet or Paneth cells. (**A**) Immunofluorescence image of HT29 cells grown for 8 days on G1 topographic surfaces. The nucleus is stained by DAPI (blue), FITC-UEA for MUC2 (green), and TRITC-labeled secondary antibodies against MUC2 antibodies (red). (**B**) Immunofluorescence images of HT29-MTX cells grown for 11 days in traditional 2D culture. The nucleus is stained by DAPI (blue), Figure 1. *N*-acetylgalactosamineα) (green), and TRITC-labeled secondary antibodies against lysozyme antibodies (red). Scale bars: 50 μm.

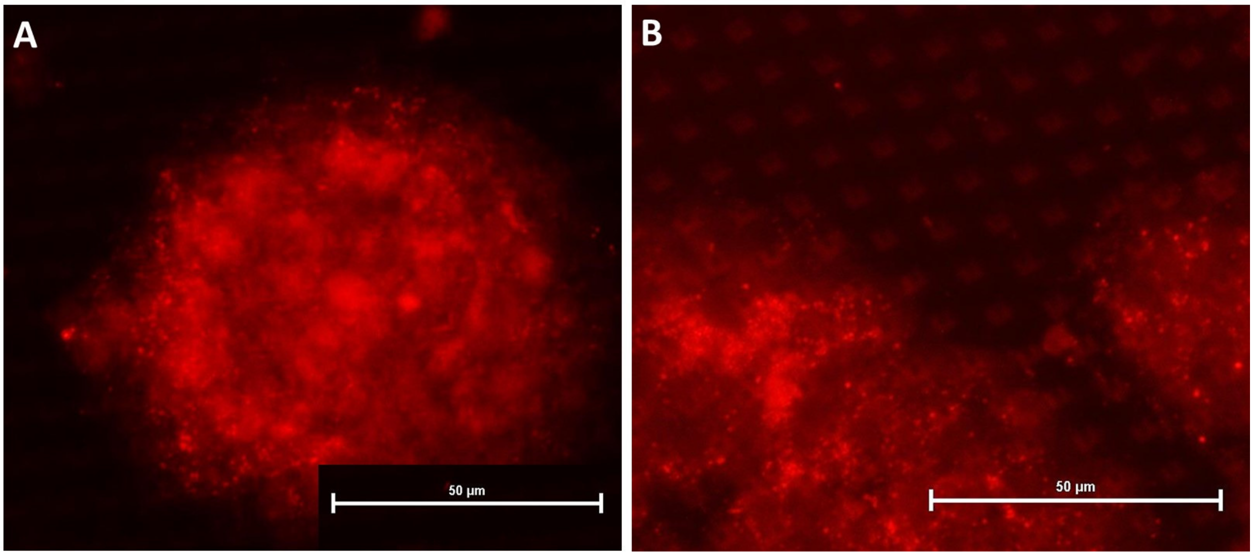

Figure S7. Magnified images of cell clusters on topographic surfaces immunostained for lysozyme. Epifluorescence of HT29 after 8 days on (**A**) G0*Hex* and (**B**) G1*Hex* stained with an antibody for lysozyme and a secondary TRITC-labeled antibody. Scale bar: 50 μm.
